# Eukaryotic translation initiation factor 3 (eIF3) subunit e is essential for embryonic development and cell proliferation

**DOI:** 10.1002/2211-5463.12482

**Published:** 2018-07-05

**Authors:** Daichi Sadato, Tomio Ono, Saki Gotoh‐Saito, Naoki Kajiwara, Namiko Nomura, Masako Ukaji, Liying Yang, Kenji Sakimura, Youichi Tajima, Keisuke Oboki, Futoshi Shibasaki

**Affiliations:** ^1^ Department of Molecular Medical Research Tokyo Metropolitan Institute of Medical Science Japan; ^2^ Department of Applied Biological Science Faculty of Science and Technology Tokyo University of Science Noda Chiba Japan; ^3^ Center for Basic Technology Research Tokyo Metropolitan Institute of Medical Science Japan; ^4^ Department of Cellular Neurobiology Brain Research Institute Niigata University Japan

**Keywords:** eIF3e, embryonic development, eukaryotic translation initiation factor 3, gene‐targeted mice, haploinsufficiency, Int6, translation

## Abstract

Mammalian eukaryotic translation initiation factor 3 (eIF3) is the largest complex of the translation initiation factors. The eIF3 complex is comprised of thirteen subunits, which are named eIF3a to eIF3 m in most multicellular organisms. The *eIF3e* gene locus is one of the most frequent integration sites of mouse mammary tumor virus (MMTV), which induces mammary tumors in mice. MMTV‐integration events result in the expression of C‐terminal‐truncated eIF3e proteins, leading to mammary tumor formation. We have shown that tumor formation can be partly caused by activation of hypoxia‐inducible factor 2α. To investigate the function of eIF3e in mammals, we generated eIF3e‐deficient mice. These *eIF3e* ^*−/−*^ mice are embryonically lethal, while *eIF3e* ^*+/−*^ mice are much smaller than wild‐type mice. In addition, *eIF3e* ^*+/−*^ mouse embryonic fibroblasts (MEFs) contained reduced levels of eIF3a and eIF3c subunits and exhibited reduced cellular proliferation. These results suggest that eIF3e is essential for embryonic development in mice and plays a role in maintaining eIF3 integrity.

**Abbreviations**
eIF3: Eukaryotic initiation factor 3
Int6: Integration site 6
MEF: mouse embryonic fibroblast
MMTV: mouse mammary tumor virus

Mammalian eIF3 is the largest complex of the translation initiation factors, with a molecular weight of around 800 kDa 1, 2. eIF3 is required for stabilizing 43S pre‐initiation complex and binding of Met‐tRNA_i_^Met^ and mRNA species to the 40S ribosome through an mRNA 5ʹ end‐dependent or end‐independent manner 3, 4, 5. Furthermore, it has been reported that eIF3 orchestrates not only translational activation but also repression via binding to the distinct stem‐loop structure localized within the 5ʹ‐untranslated region (5ʹUTR) of specific mRNA species, which could be targeted to control carcinogenesis 6. In nearly all multicellular organisms, the eIF3 complex consists of 13 subunits called eIF3a to eIF3 m 1, 7. Enormous efforts using a biochemical constitutive approach have provided a comprehensive view of the function for each eIF3 subunit 8, 9, 10, whereas the knowledge of their roles *in vivo* is still limited 11, 12, 13.

The *eIF3e* gene locus, also called integration site 6 (Int6), is one of the frequent integration sites of mouse mammary tumor virus (MMTV), which provokes mammary tumor in mice 14. MMTV‐integration events occur within intronic regions of eIF3e, resulting in generation of a C‐terminal‐truncated eIF3e/MMTV viral long terminal repeat chimeric mRNA 15. Expression of the truncated *eIF3e* is believed to have cancer‐promoting activity 16, 17.

The functions of eIF3e *in vivo* have been studied in many model organisms, and different functional aspects of eIF3e have been elucidated 18, 19, 20, 21, 22, 23. In *Schizosaccharomyces pombe*, eIF3e‐null cells are viable but exhibit a slow‐growth phenotype 18, 19, 20. *Saccharomyces cerevisiae* do not have a gene encoding eIF3e, but have structurally similar protein Pci8p that does not act as a translational regulator 21. In *Drosophila melanogaster*, eIF3e is an essential gene for survival of somatic, germline, and embryonic cells 22, by regulating cullin neddylation. In *Danio rerio*, loss of *eIF3e* leads to abnormal development, and eIF3e was identified as a tissue‐specific modulator of MEK‐ERK signaling 23.

eIF3e is a multifunctional protein involved in multiple molecular processes. It has been reported to play a role in translation 19, 24, mitosis 19, 25, nonsense‐mediated mRNA decay 26, ubiquitin‐mediated proteolysis in *S. pombe* 27, and Nedd8‐mediated proteolysis in *D. melanogaster* 22. Accordingly, eIF3e interacts with multiple proteins that constitute functional protein complexes, including a ribosomal complex, a proteasome, a COP9 signalosome 28, and a proteasome–ribosome supercomplex (translasome) 29.

We have previously demonstrated that an interaction between eIF3e and hypoxia‐inducible factor 2α (HIF2α) is important for the regulation of HIF2α degradation, independent of pVHL which is an oxygen‐dependent E3 ubiquitin ligase 30. We have also reported that eIF3e regulates angiogenesis of normal arteries and veins in mice 31. To further investigate the function of eIF3e in mice, we disrupted the *eIF3e* open reading and found that *eIF3e* ^*−/−*^ mice are embryonically lethal and *eIF3e* ^*+/−*^ mice show haploinsufficiency with concomitant decrease in the protein level of eIF3a and eIF3c subunits and reduced levels of cellular proliferation.

## Materials and methods

### Generating eIF3e‐targeted mice

Mice were maintained under specific pathogen‐free conditions. All animal (approved number: #16063) and gene recombination (approved number: #15‐040) experiments were performed in accordance with the Research Ethical Committee Guidelines of the Tokyo Metropolitan Institute of Medical Science. Targeting vector (PG00188_Y_2_D09) for the generation of reporter‐tagged null allele 32 was purchased from the European Conditional Mouse Mutagenesis Program. The vector was linearized with *Asi*SI and was electroporated into embryonic stem RENKA cells derived from C57BL/6N mice 33. G418‐resistant colonies were expanded and screened for homologous recombination by performing PCR with two specific primer pairs (one for detecting 3ʹ *loxP* sequence [forward: 5ʹ‐GAGATGGCGCAACGCAATTA‐3ʹ and reverse: 5ʹ‐GCGCCCTGTGCACAGGATGT‐3ʹ] and another for 3ʹ *loxP* long PCR [forward: 5ʹ‐GAGATGGCGCAACGCAATTA‐3ʹ and reverse: 5ʹ‐TTATGTGCTGGTCAGCAAGC‐3ʹ]). Positive clones were checked for unexpected integration of the targeting vector by southern blotting. Southern blotting was performed using DIG DNA Labeling and Detection Kit (Sigma‐Aldrich, MO, USA) according to the manufacturer's protocol. Genomic DNA (10 μg) was digested overnight with *Pac*I and was separated on a 0.7% agarose gel. Digested fragments were transferred to a positively charged nylon membrane (Sigma‐Aldrich) and were hybridized with a probe. DIG present in the probe was detected using an anti‐DIG alkaline phosphatase‐conjugated antibody and by exposing the membrane to Fujifilm LAS 4000 imager (GE Healthcare, PA, USA). Two independently isolated *eIF3e* ^*+/−*^ ES clones were individually aggregated with diploid 8‐cell embryos of Crl:CD1 (ICR). The resultant chimeras were mated to C57BL/6N mice to obtain offspring.

### Genotyping

Offspring genotypes were determined by performing PCR with two primer sets, using genomic DNA purified from tail‐tip biopsies (one set for detecting the mutant allele [forward: 5ʹ‐GTCGAGATATCTAGACCCAG‐3ʹ and reverse: 5ʹ‐GCGCCCTGTGCACAGGATGT‐3ʹ] and another set for detecting the wild‐type allele [forward: 5ʹ‐GTCTATTGTACTTGAAGCTC‐3ʹ and reverse: 5ʹ‐GCGCCCTGTGCACAGGATGT‐3ʹ]).

### Copy number assay

For copy number assay, amnion or placenta obtained from pregnant mice after euthanasia was treated with proteinase K to purify genomic DNA. Real‐time PCR was performed using 100 ng purified genomic DNA as a template and the following primers: (a) *eIF3e* intron sequence forward (5ʹ‐GCGGACGCCTTATAACCTG‐3ʹ) and *eIF3e* intron sequence reverse (5ʹ‐AGCACTGGGGTTGGTTCTC‐3ʹ) or (b) *LacZ* forward (5ʹ‐TTTCAGCCGCGCTGTACT‐3ʹ) and *LacZ* reverse (5ʹ‐CGTAGGTAGTCACGCAACTCG‐3ʹ). Copy numbers were calculated as a ratio of *LacZ* to *eIF3e*.

### Mouse embryonic fibroblast and siRNA‐based gene silencing

Mouse embryonic fibroblasts (MEFs) were prepared from E13.5 embryos obtained from heterozygous intercrosses using standard trypsinizing methods. Cells were cultured as described previously 30. Cells at passage 4 were used for further analyses. The following siRNA species directed against murine eIF3e were used: si219, 5ʹ‐AAGAACCACAGUUGUUGCGCA‐3ʹ; sim1, 5ʹ‐GACUACUGCCGUCAUAACCAACA‐3ʹ; sim2, 5ʹ‐GUCCACAUAUUCUACGCUAUUG‐3ʹ; and siCont as a negative control, 5ʹ‐GUACCGCACGUCAUUCGUAUC‐3ʹ (RNAi Co. Tokyo, Japan). MEFs were transfected with these siRNA species at 10 nm for 72 h using Lipofectamine RNAi Max (Thermo Fisher Scientific, MA, USA) according to the manufacturer's instructions.

### Western blotting

Western blotting was performed as described previously 31. Primary antibodies are listed in Table S1.

### Histology

For histological evaluation, embryos were fixed in 4% paraformaldehyde in PBS. Next, 6‐μm sections of paraffin‐embedded tissue samples were stained with hematoxylin and eosin. Anti‐E‐cadherin and antivimentin antibodies were used for immunostaining. Embryo sections were incubated with the primary antibody overnight at 4 °C and then with the biotin‐conjugated secondary antibody for 2 h at room temperature. The color was developed using DAB‐Ni. Nuclei were counterstained with Kernechtrot (Merck Millipore, Darmstadt, Germany). The stained samples were photographed using a BZ‐9000 microscope (Keyence, Osaka, Japan). All antibodies are listed in Table S1.

### Quantitative reverse‐transcription PCR (qRT‐PCR)

Total RNA isolation and qRT‐PCR were performed as described previously 31 with gene‐specific primer pairs listed in Table S2. Target gene expression was normalized to that of *GAPDH* for mice tissues or to that of *18S rRNA* gene for MEFs. Relative expression values from real‐time PCR were calculated using CFX Manager (Bio‐Rad, Hercules, CA, USA).

### Cell proliferation and translation activity assay

To evaluate cell proliferation, 5 × 10^4^ MEFs were seeded in a 12‐well plate and were harvested at indicated times after passaging. Dead cells were detected by staining with trypan blue, and the number of live and dead cells was counted at indicated times.

Total translation activity was determined by performing Coomassie Brilliant Blue (CBB) staining of total proteins separated by SDS/PAGE. Briefly, 1 × 10^6^ MEFs were counted and lysed in RIPA buffer. Equal volume of lysates was resolved by SDS/PAGE, and the gel was stained with CBB. Images of the gels were obtained using a LAS 4000 imager.

Methionine incorporation for the detection of nascent protein synthesis was evaluated using Click‐it L‐homopropargylglycine (HPG) Alexa Fluor Protein Synthesis Assay Kit (Thermo Fisher Scientific) according to the manufacturer's protocol. HPG is a methionine analogue that contains an alkyne moiety that can be fed to cultured cells and incorporated into proteins during active protein synthesis. MEFs were incubated for 30 min in methionine‐free medium supplemented with HPG. Free HPG was removed by washing with PBS, and HPG incorporated into newly synthesized proteins was labeled with Alexa Fluor 488. The incorporated fluorescence of HPG/Alexa Fluor 488 was detected using a flow cytometer (FACSCanto II; BD Biosciences, San Jose, CA, USA).

### Generation and transduction of retroviruses

HA‐tagged murine eIF3e, eIF3e‐ΔC 31, and AcGFP were subcloned into the *Not*I‐*Bam*HI site of the pQCXIP vector (TAKARA, Shiga, Japan) and were cotransfected with into GP2‐293 cells along with pVSV‐G to produce viral particles. Retroviral supernatants were harvested 48 h after transfection and concentrated by PEG centrifugation. After filtration by Millex‐HV filter (Merck Millipore), these retroviral supernatants were used to transduce genes into MEFs.

### Pulse‐chase experiments

Newly synthesized proteins were labeled using L‐azidohomoalanine (AHA), which is an analogue of methionine that contains an azide moiety. After pre‐incubation in methionine‐free medium for 30 min, MEFs were incubated for 180 min in methionine‐free medium supplemented with AHA. Free AHA was removed by washing with PBS, and complete medium was added to the MEF cultures. After incubation for 0, 24, 48, 72, and 96 h, MEFs were harvested and lysed with 1% SDS in 50 mm Tris/HCl (pH8.0). AHA incorporated into nascent proteins was labeled with biotin–alkyne by a ‘click’ reaction. Biotinylated lysates (100 μg) and streptavidin beads (JSR, Tokyo, Japan) were incubated at 4 °C for 1 h in a solution containing 0.5% NP‐40, 20 mm Tris/HCl (pH 7.4), 150 mm NaCl, and 0.5 mm DTT. Beads were washed with the same buffer three times, and pull‐down products were eluted by heating with SDS/PAGE sample buffer to perform western blotting.

### Statistical analysis

Statistical analysis was performed by GraphPad Prism (Graph Pad Software, CA, USA). Data are expressed as means ± standard deviation. Differences were evaluated by two‐way ANOVA followed by Bonferroni's post hoc multiple comparison for time‐course data or by two‐tailed Student's *t*‐test for the case of comparisons of two‐group data.

## Results

### Expression profiles of mouse eIF3e

**Figure 1:**
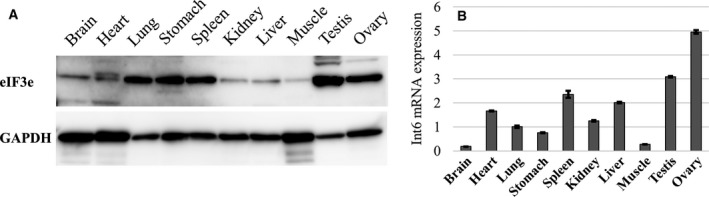
Expression profiles of eIF3e mRNA and protein in mice. Cell lysates and cDNA species were prepared from 10‐week‐old male C57BL/6N mice. Only ovary samples were prepared from female mice of the same age. (A) Protein expression patterns of eIF3e (upper panel) and control GAPDH (lower panel) in different mouse organs. (B) mRNA expression patterns of *eIF3e* in different mouse organs. eIF3e expression was normalized using GAPDH expression; columns represent mean values with SD.

We examined eIF3e expression in adult mouse organs by performing western blotting and qRT‐PCR (Fig. 1). Results of western blotting showed a ubiquitous expression pattern with variable expression levels. High eIF3e protein and mRNA expression were found in the testis, ovaries, and spleen (Fig. 1A,B). Discordant expression levels between mRNA and its protein were observed in the heart, lung, stomach, kidneys, and liver.

### Targeted disruption of eIF3e

**Figure 2:**
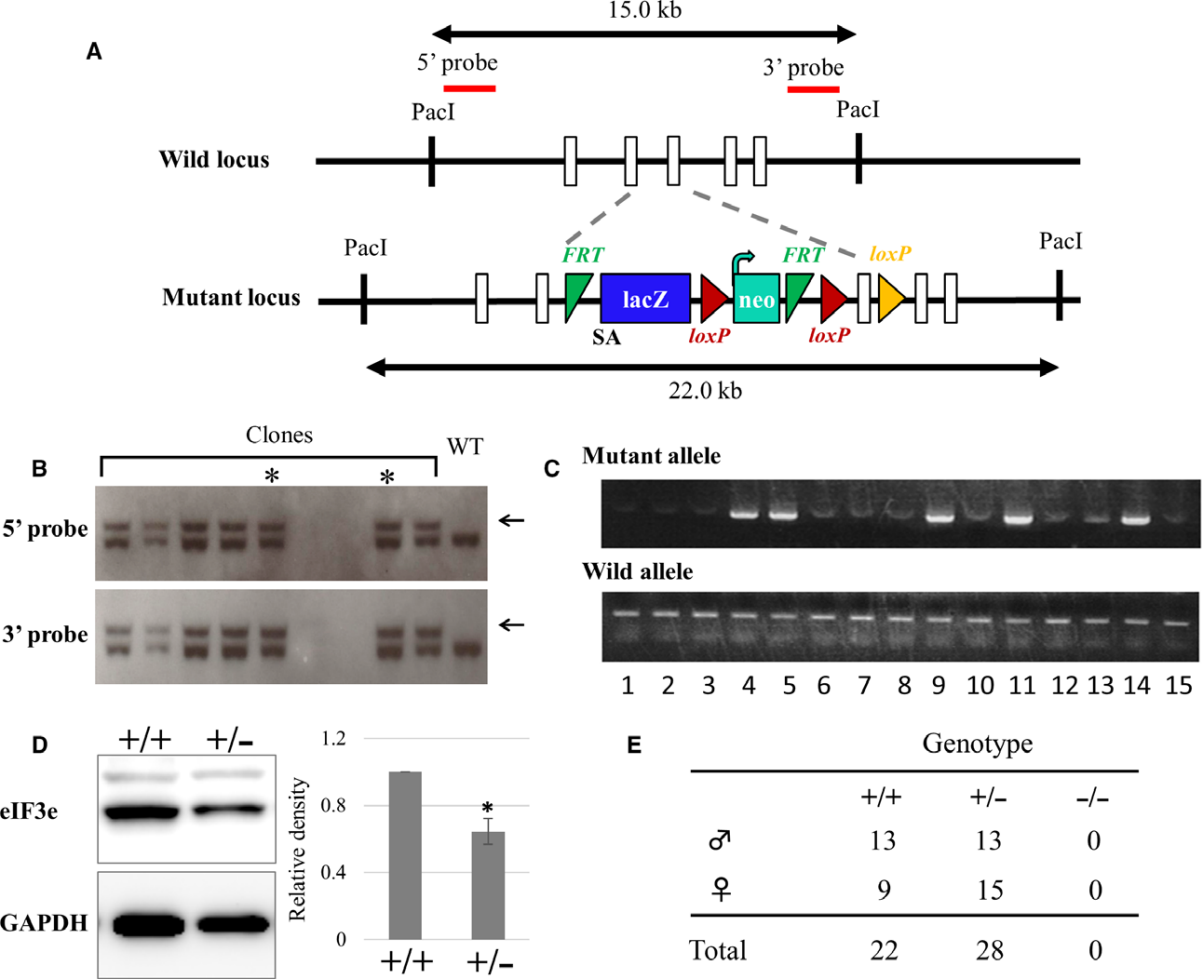
Targeted disruption of mouse *eIF3e*. (A) Scheme for generating *eIF3e‐*deficient mice. The genomic structure of *eIF3e* and the desired homologous recombination allele along with southern blotting probes are shown. Open boxes represent eIF3e exons, and the other functional sequences or cassettes are illustrated with their names. SA refers to the splicing acceptor for expressing lacZ and that modifies the mRNA structure of *eIF3e* for target disruption. Dotted lines show the insertion region that results in desired recombination. Numerical value (kb) with double arrows indicates genomic fragment size digested with *Pac*I. (B) Representative results of southern blotting of ES cell clones with 5ʹ (upper panel) and 3ʹ probes (lower panel). Arrows indicate the target allele band. The asterisk indicates the clones used for generating chimeric mice. (C) Representative results of PCR genotyping of tail DNA of offspring obtained from heterozygous intercrosses. (D) Western blotting of eIF3e in the liver lysates of *eIF3e* ^+/+^ (+/+) and *eIF3e* ^+/−^ (+/−) mice, which is representative of four animals of each genotype analyzed. GAPDH was used as a loading control. Columns represent mean values with SD. Asterisk indicates *P* < 0.05 when compared to *eIF3e* ^+/+^. (E) Genotyping of mice generated from heterozygous intercrosses.

To investigate the role of eIF3e in mice, we disrupted the *eIF3e* allele as shown in Fig. 2A. Results of PCR with two primer sets (Fig. S1A) for the first (Fig. S1B) and second screening (Fig. S1C) showed that 14% (20/144) G‐418‐resistant ES cell clones had at least the 3ʹ region of the targeting cassette. Twenty positive clones were further verified by southern blotting (Fig. 2B), and two independent *eIF3e* ^+/−^ ES cell clones were chosen for further study. We mated the chimeric mice with wild‐type mice and obtained *eIF3e* ^*+/−*^ mice in their offspring. The expression of eIF3e was decreased in the liver of *eIF3e* ^+/−^ mouse, compared with that of *eIF3e* ^+/+^ mouse, which likely depends on gene dosage (Fig. 2D). *eIF3e* mRNA levels were also decreased in *eIF3e* ^+/−^ MEFs compared to that in *eIF3e* ^*+/+*^ MEFs (Fig. 4C). In the *eIF3e* ^+/−^ lane, eIF3e proteins were detected at 50 kDa without additional smaller band (data not shown). This suggests that the disruption mutation resulted in a null‐like state. *eIF3e* ^+/−^ mice were crossed to produce *eIF3e* ^−/−^ mice. Genotyping of 50 offspring from 14 litters showed that 22 offspring were wild‐type (*eIF3e* ^+/+^) and 28 were heterozygous (*eIF3e* ^+/−^) in the targeted allele (Fig. 2E). Homozygous (*eIF3e* ^−/−^) offspring were not detectable. The average size of litter produced from heterozygous intercrosses (3.0 ± 0.8 mice/litter) was smaller than those from wild‐type and heterozygous intercrosses (7.0 ± 1.9 mice/litter). These results suggest that *eIF3e* is gene essential for embryogenesis in mice.

### eIF3e deficiency causes early embryonic lethality

In E13.5 embryos from *eIF3e* ^+/−^ intercrosses, we isolated measurable embryos and a few very small fetal membranes that were considered a vestige of embryo (Fig. S2A). To clarify the genotypes of these embryos, we calculated the dosage of the *LacZ* gene derived from the gene‐targeting vector, using quantitative PCR with genomic DNA as described in Fig. S2B. *LacZ*/wild‐type allele ratios likely showed the genomic *LacZ* copy numbers, that is, 0, 1, and 2, which correspond to −/−, +/−, and +/+ of the embryo genotypes, respectively (Fig. S2C).

**Figure 3:**
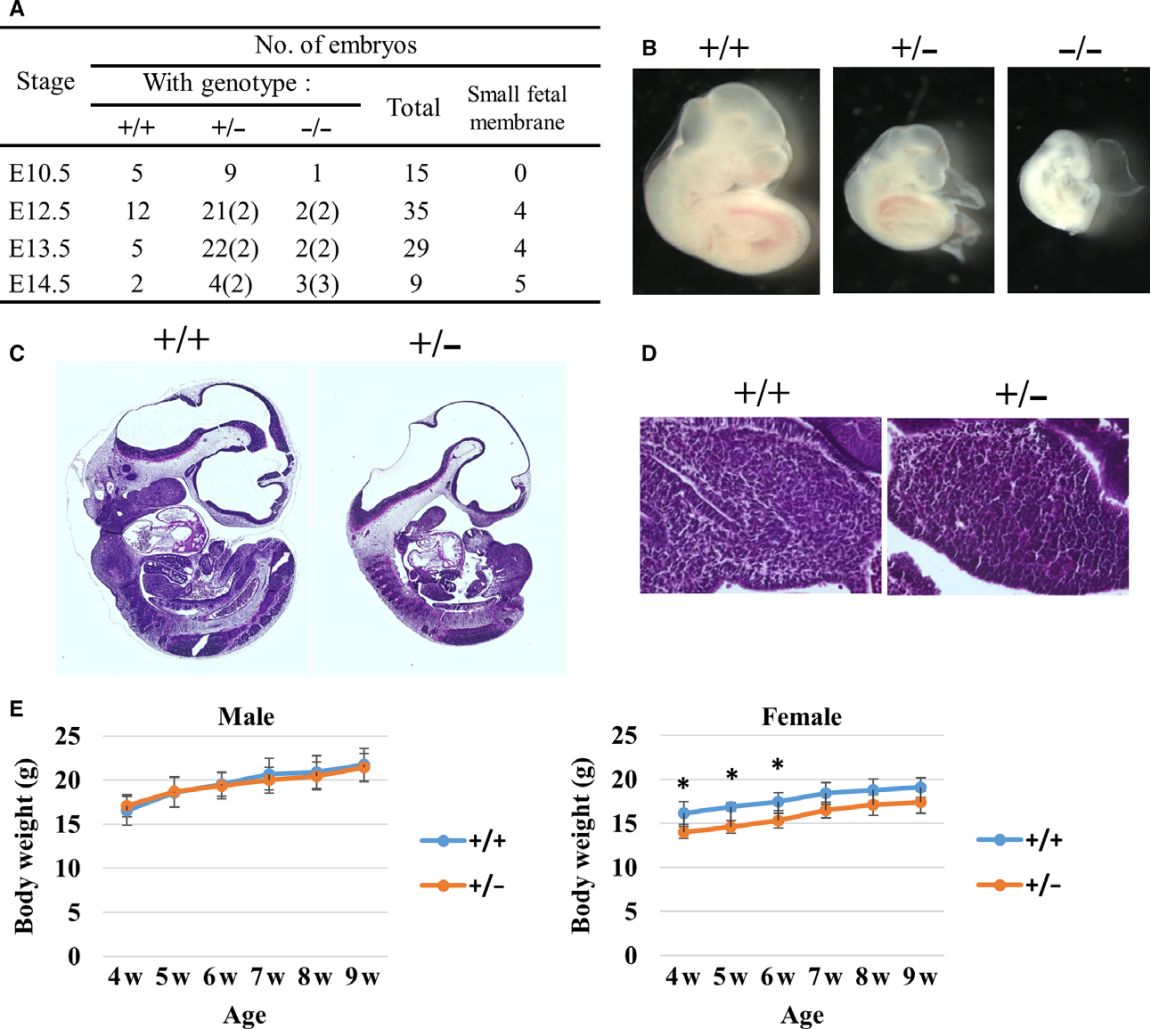
Essential role of *eIF3e* in mouse embryonic development. (A) Genotyping and phenotyping analyses of embryos obtained from heterozygous intercrosses. The number of abnormal small fetal membranes is indicated in parentheses. (B) Representative images of *eIF3e* ^+/+^, *eIF3e* ^+/−^, and *eIF3e* ^−/−^ embryos at E10.5. Hematoxylin and eosin staining of E10.5 whole embryos (C) and a magnified image of fetal liver (D). (E) Time‐course change of weight from weaning (4 weeks) to 9 weeks of age. Body weights shown by points represent mean values with SD. (*two‐way ANOVA for age/genotype; *P* < 0.001, followed by Bonferroni's post hoc multiple comparison test; *P* < 0.05, *n* = 5 for each group).

To understand to what extent the *eIF3e* ^−/−^ embryo can develop *in utero*, we obtained E10.5 to 14.5 embryos from *eIF3e* ^+/−^ intercrosses. As summarized in Fig. 3A, at E10.5, the frequency of *eIF3e* ^−/−^ embryos (0.6%) was lower than that from the expected Mendelian ratio (25%). Interestingly, these embryo sizes were likely consistent with their genotypes (*eIF3e* ^+/+^ >* eIF3e* ^+/−^ >* eIF3e* ^−/−^) (Fig. 3B). At E12.5 to E14.5, there were no *eIF3e* ^−/−^ embryos with preserved normal size and shape, and we found the abnormal small fetal membranes whose genotypes were *eIF3e* ^−/−^ or *eIF3e* ^+/−^. These results suggest that embryonic lethality of *eIF3e* ^−/−^ fetuses might be explained by antecedent growth retardation and/or resorbing of embryos started before E10.5.

Sagittal sections of *eIF3e* ^+/+^ and *eIF3e* ^+/−^ embryos at E10.5 were prepared to test whether heterozygous *eIF3e* deletion led to tissue abnormality. *eIF3e* ^+/−^ embryos clearly showed hypoplasia (Fig. 3C). The organ size of *eIF3e* ^+/−^ embryonic liver was smaller than *eIF3e* ^+/+^. However, a cell size of embryonic liver was not different between *eIF3e* ^+/+^ and *eIF3e* ^+/−^ (Fig. 3D). Thus, smaller‐size *eIF3e* ^+/−^ embryos were likely caused by growth retardation without morphological abnormalities. Indeed, *eIF3e* ^+/−^ pups were grown to be fertile, but the body weight of female *eIF3e* ^+/−^ pups is significantly lower than that of *eIF3e* ^+/+^ from weaning to 9 weeks of age (Fig. 3E), although the mechanisms that underlie the sex‐biased phenotype have remained elusive. These results suggest that *eIF3e* ^+/−^ show haploinsufficiency in murine normal development.

We next examined whether decreased eIF3e levels affect the epithelial‐to‐mesenchymal transition (EMT) in the developing mouse embryo, because it has been reported that eIF3e acts as an EMT regulator in some epithelial cell lines 34, 35. We stained paraffin sections of *eIF3e* ^+/+^ and *eIF3e* ^+/−^ embryos at E10.5 with antivimentin and anti‐E‐cadherin antibodies as a mesenchymal and epithelial marker, respectively. The developmental stages of *eIF3e* ^+/+^ and *eIF3e* ^+/−^ were not completely synchronized owing to growth retardation of *eIF3e* ^+/−^ embryos. Nonetheless, vimentin and E‐cadherin signals were comparable between *eIF3e* ^+/+^ and *eIF3e* ^+/−^ (Fig. S3A,B). To further examine this in primary cells, several mesenchymal markers as well as E‐cadherin expression were determined in MEFs by western blotting. The expression of each marker was not significantly changed between *eIF3e* ^+/+^ and *eIF3e* ^+/−^ MEFs, suggesting that it is unlikely to result in EMT in *eIF3e* ^+/−^ mice (Fig. S3C).

### Heterozygous deletion of eIF3e inhibits normal cell proliferation

**Figure 4:**
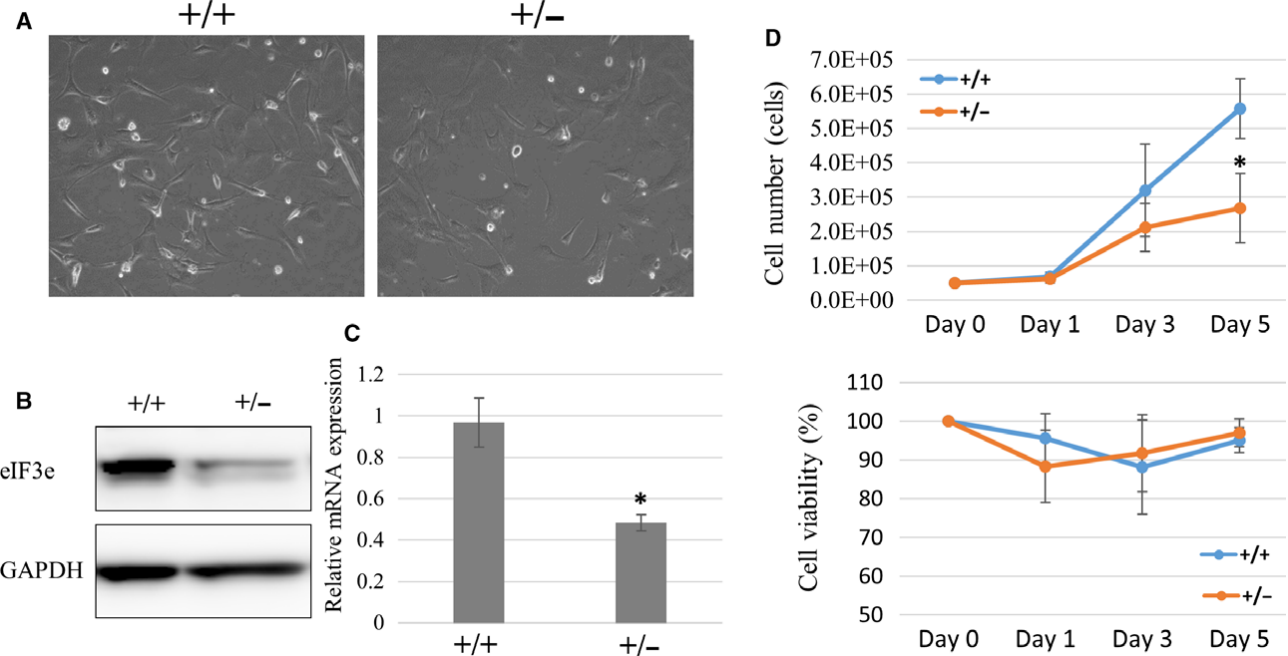
Impaired proliferation of *eIF3e* ^+/−^ cells. (A) Microscopy images of MEFs established from *eIF3e* ^+/+^ (+/+), *eIF3e* ^+/−^ (+/−) mice embryos. (B) Western blotting of eIF3e and GAPDH as a loading control (left) and (C) qRT‐PCR showing the eIF3e mRNA expression normalized by *18S rRNA* gene (right) in the *eIF3e* ^+/+^ and *eIF3e* ^+/−^ MEFs. Columns represent mean values with SD from two independent experiments. (D) Cell numbers and viability of *eIF3e* ^+/+^ and *eIF3e* ^+/−^ MEFs from three clones each. Cell numbers are counted at 1, 3, and 5 days after starting to culture cells at 5 × 10^4^. Viabilities (%) calculated by trypan blue exclusion test are shown at 1, 3, and 5 days. Columns represent mean values with SD from three independent experiments. The asterisk indicates *P* < 0.05 when compared to *eIF3e* ^+/+^.

For an analysis at the cellular level, we established mouse embryonic fibroblasts (MEFs) from *eIF3e* ^+/+^ and *eIF3e* ^*+/−*^ fetuses. We could not establish MEFs from *eIF3e* ^−/−^ embryos. Cultured *eIF3e* ^+/+^ and *eIF3e* ^*+/−*^ MEFs showed comparable size and morphology (Fig. 4A). Next, we measured the level of protein and mRNA expression of eIF3e in these MEFs. The protein and the mRNA expression of eIF3e was decreased in *eIF3e* ^*+/−*^ MEFs (Fig. 4B,C) compared to those of *eIF3e* ^*+/+*^ MEFs. To evaluate cell proliferation and cell viability of both genotypes, we cultured *eIF3e* ^*+/+*^ and *eIF3e* ^*+/−*^ MEFs for 5 days (Day 1 to 5). *eIF3e* ^+/−^ MEFs showed significantly lower proliferation than *eIF3e* ^+/+^ MEFs (*P* < 0.001, Fig. 4D, upper panel). However, cell viabilities were not different between *eIF3e* ^+/+^ and *eIF3e* ^*+/−*^ MEFs (Fig. 4D, lower panel), indicating that the deletion of *eIF3e* makes cell proliferation slower, independent of cell death.

### Heterozygous deletion of eIF3e affects normal translation

**Figure 5:**
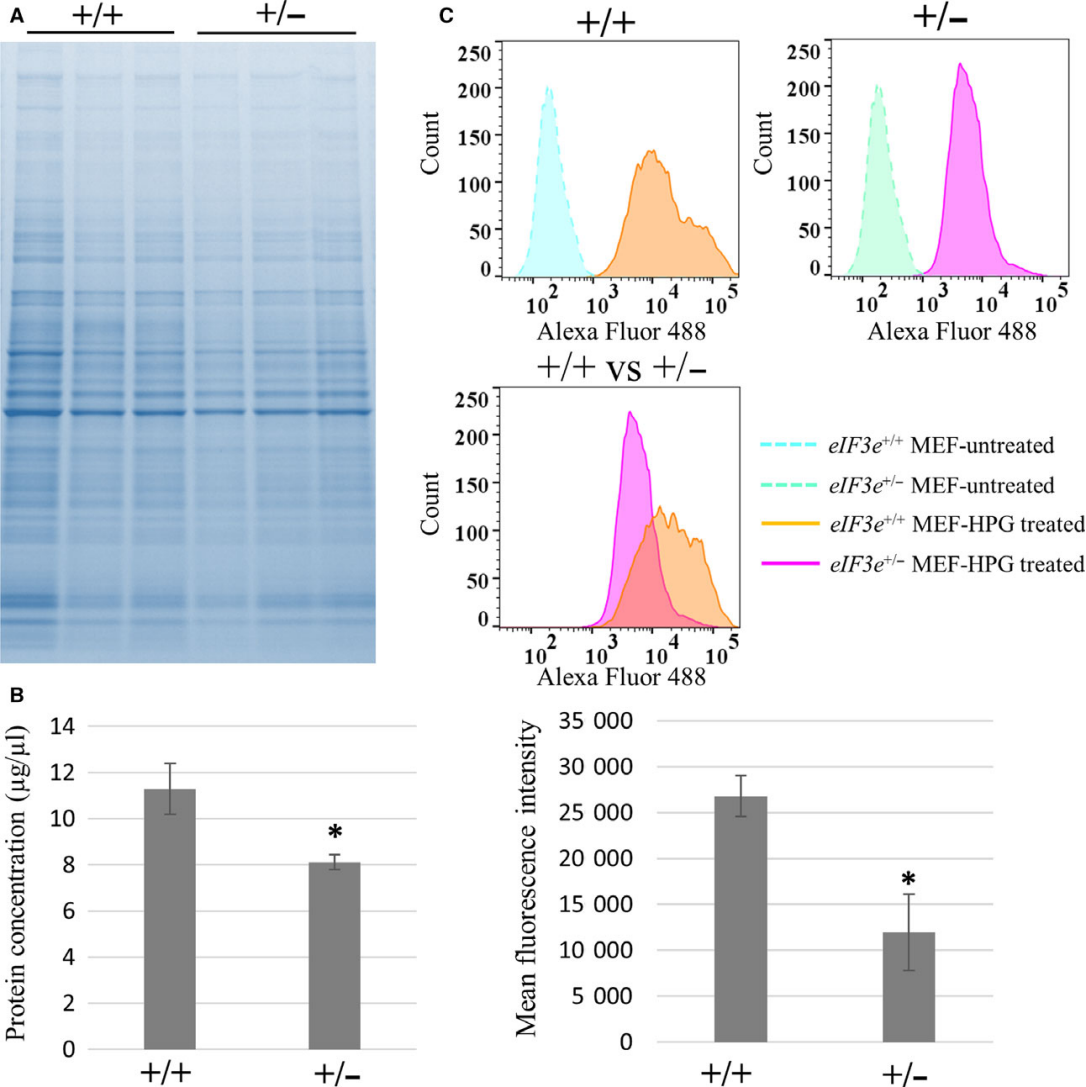
Decrease in translational activity in *eIF3e* ^+/−^ cells. (A) Total protein level of *eIF3e* ^+/+^ and *eIF3e* ^+/−^ MEFs. Each lane represents a sample from an individual animal. Protein lysates were visualized by CBB staining. (B) Protein concentrations of MEF lysates were measured by the BCA method. Columns represent mean values with SD from three independent sets of three MEF clones. (C) HPG incorporation of *eIF3e* ^+/+^ and *eIF3e* ^+/−^ MEFs demonstrated by FACS. FACS histograms are representative of three independent experiments. Histogram representing HPG‐treated and HPG‐non‐treated cells are shown in the upper panel, and HPG‐treated *eIF3e* ^+/+^ MEFs (purple) and *eIF3e* ^+/−^ cells (orange) in the middle panel. Bar graph shows the mean fluorescence intensity values for HPG‐incorporated cells (mean ± SD) from three independent FACS experiments. The asterisk indicates *P* < 0.05 when compared to *eIF3e* ^+/+^.

Next, to examine whether protein biogenesis is influenced by heterozygous deletion of *eIF3e*, we compared *eIF3e* ^*+/−*^ and *eIF3e* ^*+/+*^ MEFs. We independently established three MEFs in each genotype derived from the fetuses. Equal number of MEFs were lysed and separated by SDS/PAGE, and CBB staining was performed to visualize the total amount of protein. Results of CBB staining showed that *eIF3e* ^+/−^ MEF proteins slightly decreased compared to those of *eIF3e* ^+/+^ MEF (Fig. 5A). To confirm this, we also measured the protein concentration of these lysates. The protein concentration of *eIF3e* ^+/−^ lysates was significantly lower than that of *eIF3e* ^+/+^, by about 20% (Fig. 5B). These suggest that heterozygous deficiency of eIF3e impairs global translational activity.

To further evaluate the insufficient protein biogenesis in *eIF3* ^*+/−*^ MEFs, we next measured methionine incorporation. *eIF3* ^*+/+*^ and *eIF3* ^*+/−*^ MEFs effectively incorporated the methionine analogue HPG labeled by Alexa Fluor 488 (orange and purple area, Fig. 5C). Mean fluorescence intensity of *eIF3e* ^+/−^ cells was significantly lower than that of *eIF3e* ^+/+^ cells (*P* < 0.05, 11960.0 ± 1698.0 vs. 26801.3 ± 915.7), suggesting that methionine incorporation was impaired in *eIF3e* ^+/−^ MEFs (Fig. 5C). These data indicate that translation speed per unit time in *eIF3e* ^+/−^ cells decreased to approximately half of that in *eIF3e* ^+/+^ cells and show that heterozygous deletion of *eIF3e* affects normal translation.

### Heterozygous deletion of eIF3e disrupts the stability of eIF3a and eIF3c subunits

**Figure 6:**
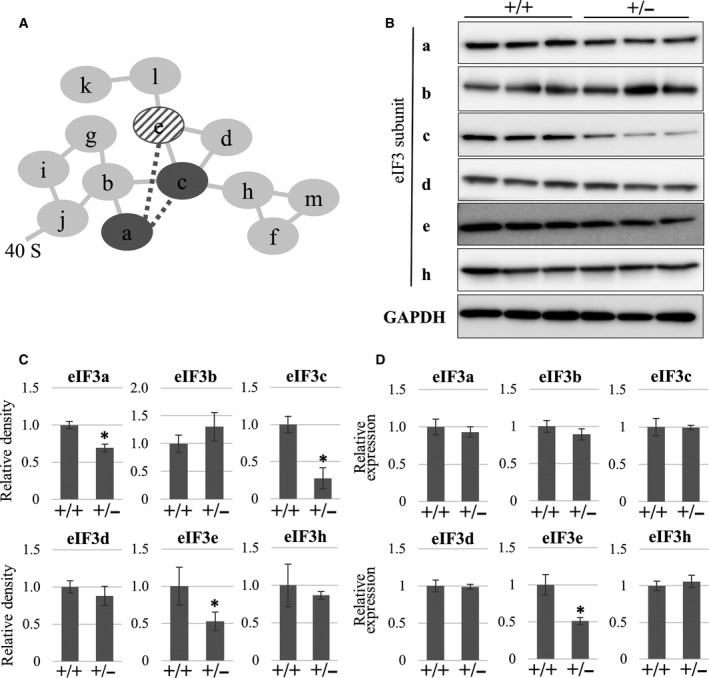
*eIF3e* deficiency selectively leads the decrease in eIF3a and eIF3c protein expression. (A) The diagram shows subunit interactions within the eIF3 complex. Each line indicates subunit–subunit interaction based on a mass‐based study (solid lines) 27 or protein‐based assay (dotted lines) 28. Dark gray circles (eIF3a and eIF3c) and a diagonal circle (eIF3e) indicate the targets affected by eIF3e disruption. (B) Western blotting of the indicated eIF3 subunits in the total cell lysates of *eIF3e* ^*+/+*^ (+/+) and *eIF3e* ^*+/−*^ (+/−) MEFs. GAPDH was used as a loading control. (C) Protein expression of the indicated eIF3 subunits. Band densities from western blot results were calculated and are shown by the columns representing the mean values with SD. (D) qRT‐PCR showing mRNA expression of the indicated eIF3 subunits. mRNA expression of each gene was normalized using that of the 18S rRNA gene. Columns represent mean values with SD from three independent experiments. The asterisk indicates *P* < 0.05 when compared to eIF3e^+/+^.

**Figure 7:**
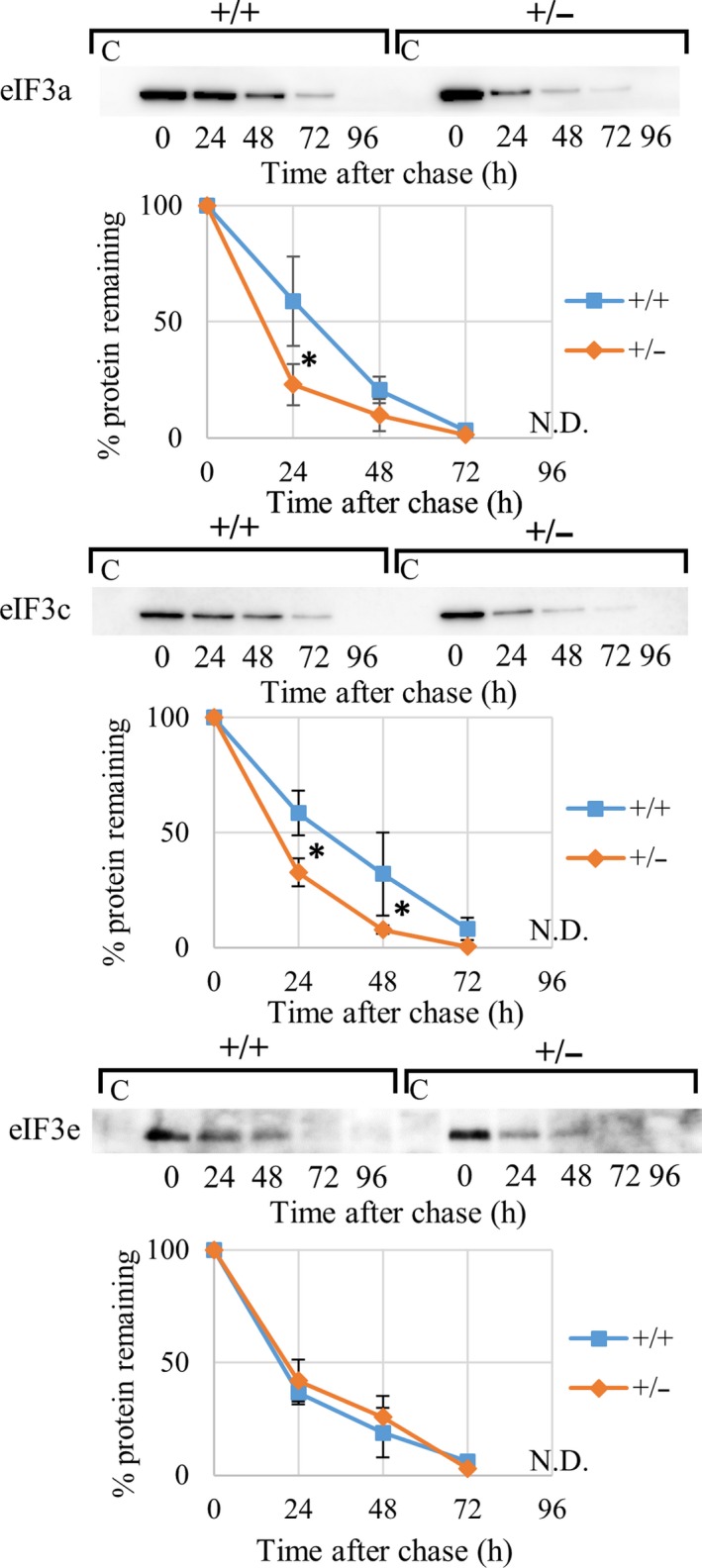
Increase in proteolytic instability of eIF3a and eIF3c in *eIF3e* ^+/−^ MEFs. Pulse‐chase analysis of eIF3a, eIF3c, and eIF3e in *eIF3e* ^*+/+*^ (+/+) and *eIF3e* ^*+/−*^ (+/−) MEFs. Nascent proteins were labeled by AHA (pulse); cells were collected at 0–96 h every 24 h after labeling (chase), and AHA‐incorporated proteins were biotinylated and pulled down using streptavidin (SA) beads to analyze by immunoblot (upper) and also perform band density analysis (lower). Lane C in the immunoblot serves as a negative control sample from SA beads incubated with nonbiotinylated lysates. Band intensities from three western blot results were calculated by assuming the highest point to be 100%. The calculated data are shown by a line chart (mean ± SD, blue as *eIF3* ^*+/+*^ and orange as *eIF3* ^*+/−*^ MEFs). Asterisks indicate level of statistical significance (*two‐way ANOVA for hour/genotype; *P* < 0.001, followed by Bonferroni's post hoc multiple comparison test; *P* < 0.05, *n* = 3–4 for each group).

To examine the effect of heterozygous deletion of *eIF3e* on the stability of the eIF3 complex (Fig. 6A), we compared the protein levels of various eIF3 subunits in *eIF3e* ^+/−^ and *eIF3e* ^+/+^ MEFs (Fig. 6B). Using western blotting and band densitometry analysis, eIF3a and eIF3c protein expressions were also reduced in *eIF3e* ^+/−^ MEFs without significant impact for the expression of other subunits (Fig. 6B,C). Given that the mRNA levels of eIF3a, eIF3b, eIF3c, eIF3e, and eIF3 h were not changed between *eIF3e* ^+/−^ and *eIF3e* ^+/+^ MEFs (Fig. 6D), the decrease in eIF3a and eIF3c proteins is not attributable to regulation at mRNA level. To determine whether altered protein stability is responsible for the reduction of eIF3a and eIF3c levels in *eIF3e* ^*+/−*^ MEFs, pulse‐chase experiments were performed and showed that eIF3a and eIF3c proteins had reduced stability in *eIF3* ^*+/−*^ MEFs compared to that in *eIF3* ^*+/+*^ MEFs, whereas eIF3e stability was unchanged (Fig. 7). These data suggest that eIF3e might stabilize eIF3a and eIF3c, and therefore, haploinsufficiency of eIF3e might be explained at least in part by instability of the eIF3 complex.

### eIF3e is necessary and sufficient for the concomitant reduction of eIF3a and eIF3c in eIF3e^+/−^ MEFs

**Figure 8:**
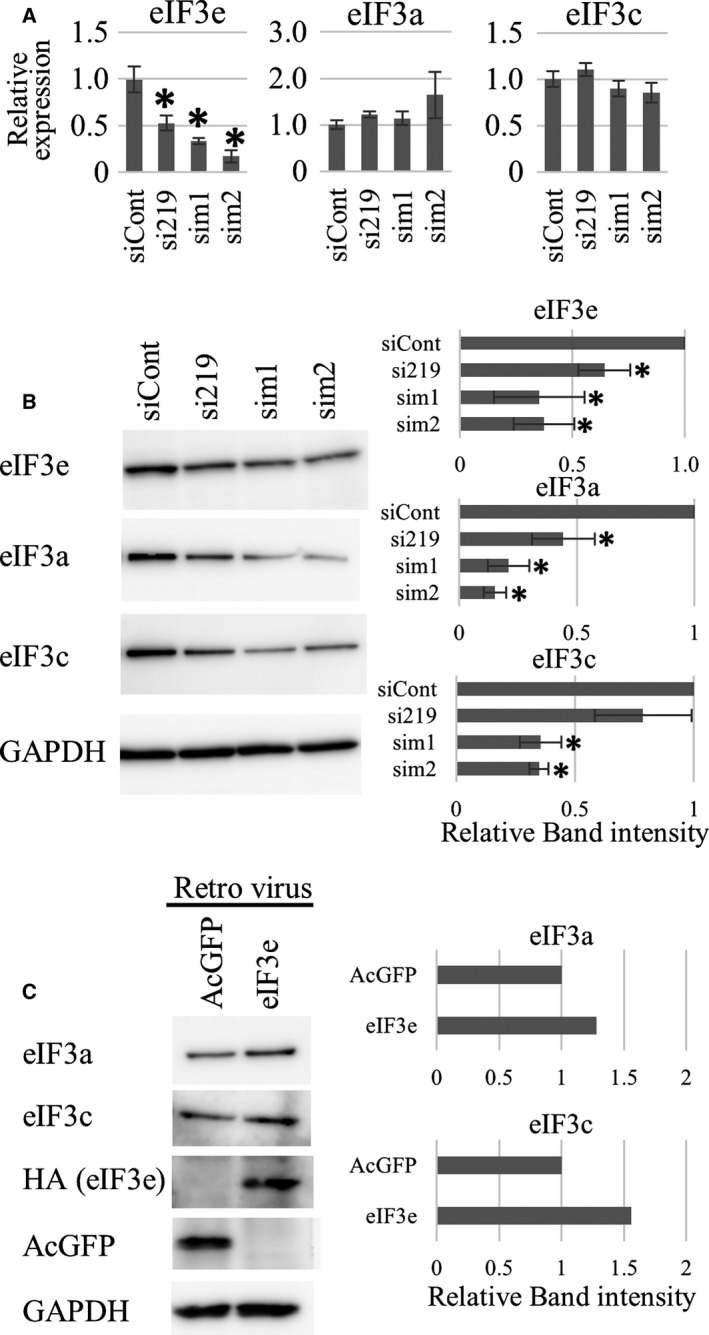
Effects of *eIF3e* silencing and forced *eIF3e* expression in *eIF3e* ^+/−^ MEFs. (A) qRT‐PCR showing mRNA expression of eIF3a, eIF3c, and eIF3e. mRNA expression of each gene was normalized to that of the *18S rRNA* gene. Columns represent mean values with SD from three independent experiments. The asterisk indicates *P* < 0.05 when compared to siCont‐transfected MEFs. (B) Western blotting of eIF3a, eIF3c, and eIF3e in the total cell lysates of wild‐type MEFs transfected with the indicated siRNA species. GAPDH was used as a loading control. Band densities from western blot results were calculated and are shown by the columns representing the mean values with SD. The asterisk indicates *P* < 0.05 when compared to siCont‐transfected MEFs. (C) Western blotting of the indicated eIF3 subunits and transgenes (HA: exogenous eIF3e; AcGFP: control) in the total cell lysates of *eIF3e* ^*+/−*^ MEFs infected with the indicated genes packaged by retroviruses. GAPDH was used as a loading control. Western blot and relative band intensities were representative of three independent experiments.

To confirm the concomitant reduction of eIF3a and eIF3c in *eIF3e* ^+/−^ MEFs, we performed gene silencing experiments in wild‐type MEFs using three siRNA species against eIF3e. The expression levels of eIF3e mRNA and protein were decreased using all three siRNA species, suggesting that these were working *in vitro* (Fig. 8A,B). Reduced protein levels were observed for eIF3a and eIF3c after treatment with eIF3e siRNA species, independent of mRNA levels of eIF3a and eIF3c (Fig. 8B). Next, to examine whether forced eIF3e expression in *eIF3e* ^+/−^ MEFs can rescue these cellular phenotypes, HA‐tagged eIF3e was retrovirally expressed in *eIF3e* ^+/−^ MEFs. Western blotting successfully detected exogenous eIF3e and AcGFP. eIF3a and eIF3c protein levels increased in *eIF3e* ^+/−^ MEFs expressing exogenous eIF3e but not in the control. These results suggest that eIF3e is necessary and sufficient for the stabilization of eIF3a and eIF3c proteins (Fig. 8C).

## Discussion

We hypothesize that fetal death in *eIF3e* ^−/−^ mice may be because global or selective translation activity is not sufficient to maintain cell growth or proliferation in embryos lacking eIF3e. In addition, we should point out that EMT might occur in eIF3e^−/−^ embryos. Embryonic lethality in *eIF3 m‐*deficient mice, which had smaller body size at E9.5 than wild‐type mice, might also be attributed to translation dysfunction 11. Additionally, mice lacking eIF3b subunit showed embryonic lethality from E3.5 to E13.5 in 12. These suggest that the deficiency of eIF3 subunits might be integrated in the arrest of global translation and thus highlighted the importance of eIF3 subunits in early embryonic development. Furthermore, analysis of *eIF3 m* ^*+/−*^ mice indicates a positive correlation between eIF3 expression levels and body weight or organ size 11. Similar results were obtained in the present study using *eIF3e* ^+/−^ mice (Fig. 3). The impaired methionine incorporation in *eIF3e* ^+/−^ cells supports our hypothesis mentioned above (Fig. 5C). Considering qualitative differences between the methionine incorporation experiment and the polysomal experiment 18, it is not fully analyzed to know how and which step of translation is exactly affected. Moreover, another possibility of nontranslational defects, including methionine transport and metabolism in eIF3e+/− cells, is not ruled out. Therefore, further analysis (i.e., polysome experiment) is needed to better characterize the cellular haploinsufficient phenotype in *eIF3e* ^+/−^ animals.

Regarding the phenotype observed for eIF3e+/− mice, we hypothesize that haploinsufficiency of human *eIF3e* gene might be a cause of an undiagnosed congenital disorder.

Results of the present study also showed that eIF3e is critical for stabilizing eIF3a and eIF3c subunits (Fig. 6). Mass spectrometry studies have suggested that eIF3e, eIF3l, and eIF3k form a subcomplex that binds to the eIF3 complex through an eIF3e–eIF3c interaction 36. Other studies have shown that eIF3e binds to an eIF3a–eIF3c heterodimer in different species 37, 38. Our results support the possibility that eIF3e binds to and stabilizes the eIF3a–eIF3c core dimer (Fig. 6A). As we focused on only a limited number of eIF3 subunits in the present study, further comprehensive analyses are required to completely understand the regulation of the eIF3 complex at protein level.

It has been reported that full‐length eIF3e is required for cell proliferation in human glioblastoma cells 39 and in *Neurospora crassa* 38. The present study in mice supports these reports. However, truncated eIF3e protein caused by MMTV‐integration has an ability of promoting transformation 17, 40. It is reasonable to think that the oncogenic mutation in *eIF3e* gene caused by MMTV‐integration is gain of function but not loss of function. Indeed, Chiluiza *et al*. have reported that truncated eIF3e causes a shift from cap‐dependent to cap‐independent translation 41. To extend our knowledge further on the roles of eIF3e, especially in cancer, we believe it important to consider classification of the loss of function or gain of function of eIF3e. Moreover, noncanonical functions of eIF3e exerted through molecular interactions with proteins, such as HIF2α 30, MCM7 42, ATM 43, or MIF4GD/SLIP1 44, have been reported, but their physiological and/or pathological importance still remains elusive. We envision that eIF3e acts as a unique modifier in several disease states.

In this study, we established an *eIF3e*‐null allele in mice. *eIF3e* ^−/−^ results in embryonic lethality at early stage of embryo, suggesting that eIF3e is essential for embryonic development. This is the first report of mice carrying the eIF3e‐null allele. We also analyzed embryos, MEFs, and mice from *eIF3e* ^+/+^ and *eIF3e* ^*+/−*^ and found that eIF3e^+/−^ shows haploinsufficiency. This suggests two copies of *eIF3e* are required for cell proliferation and normal protein biogenesis *in vivo* and *in vitro*.

## Author contributions

FS conceived this study. FS, DS, KO, and SG designed the experiments. DS, MU, NK, YT, and NN carried out the experiments. TO, LY, and KS generated the gene‐targeted mice. DS, KO, SG, and FS analyzed the data and wrote the manuscript.

## Supporting information


**Fig. S1.** Screening of Int6/eIF3e‐targeted mouse ES cells by PCR. (A) The scheme for targeted ES cell screening by performing PCR. The homologous recombination allele and the two primer sets for the first and second screening are shown. (B) Representative results of PCR genotyping with the first set of screening primers. (C) Representative results of PCR genotyping with the second set of screening primers. Arrows indicate the predicted bands, and asterisk indicates nonspecific bands.

**Fig. S2.** Copy number detection for genotyping of embryos. (A) Photo image of E13.5 embryos from *eIF3e* ^+/−^ intercrossing. (B) The genomic structure of *eIF3e* targeted homologous recombination allele, along with primers used for copy number assays, is shown. (C) Representative results of the copy number assay showing the copy number ratio (*LacZ/eIF3e* intron). Columns represent mean values with SD from triplicate samples. Asterisk indicates the sample with abnormality (extremely small or empty fetal membrane).

**Fig. S3.** Expression of epithelial and mesenchymal markers in mice embryos and MEFs. Sagittal embryo sections show immunohistochemical analysis performed by antivimentin antibody (A, shown in black) and anti‐E‐cadherin antibody (B, shown in black) in *eIF3e* ^+/+^ and *eIF3e* ^+/−^ at E10.5. (C) Western blotting of the indicated epithelial and mesenchymal marker proteins in the total cell lysates of *eIF3e* ^*+/+*^ (+/+) and *eIF3e* ^*+/−*^ (+/−) MEFs from three independent clones. GAPDH was used as a loading control. Band densities were calculated from western blot results and shown by graphs (means ± standard deviation). *P* value was calculated using an unpaired two‐tailed Student's *t*‐test.

**Table S1.** Monoclonal and polyclonal antibodies used in western blot and immunohistochemistry.
**Table S2.** Specific primers used in qRT‐PCR experiments.

## References

[1] Hinnebusch AG (2006) eIF3: a versatile scaffold for translation initiation complexes. Trends Biochem Sci 31, 553–562. 16920360 10.1016/j.tibs.2006.08.005

[2] Siridechadilok B , Fraser CS , Hall RJ , Doudna JA and Nogales E (2005) Structural roles for human translation factor eIF3 in initiation of protein synthesis. Science 310, 1513–1515. 16322461 10.1126/science.1118977

[3] Jackson RJ , Hellen CU and Pestova TV (2010) The mechanism of eukaryotic translation initiation and principles of its regulation. Nat Rev Mol Cell Biol 11, 113–127. 20094052 10.1038/nrm2838 PMC4461372

[4] Aitken CE and Lorsch JR (2012) A mechanistic overview of translation initiation in eukaryotes. Nat Struct Mol Biol 19, 568–576. 22664984 10.1038/nsmb.2303

[5] Hinnebusch AG and Lorsch JR (2012) The mechanism of eukaryotic translation initiation: new insights and challenges. Cold Spring Harb Perspect Biol 4, a011544. 22815232 10.1101/cshperspect.a011544 PMC3475172

[6] Lee AS , Kranzusch PJ and Cate JH (2015) eIF3 targets cell‐proliferation messenger RNAs for translational activation or repression. Nature 522, 111–114. 25849773 10.1038/nature14267 PMC4603833

[7] Pick E , Hofmann K and Glickman MH (2009) PCI complexes: beyond the proteasome, CSN, and eIF3 Troika. Mol Cell 35, 260–264. 19683491 10.1016/j.molcel.2009.07.009

[8] Silvera D , Formenti SC and Schneider RJ (2010) Translational control in cancer. Nat Rev Cancer 10, 254–266. 20332778 10.1038/nrc2824

[9] Bandyopadhyay A , Lakshmanan V , Matsumoto T , Chang EC and Maitra U (2002) Moe1 and spInt6, the fission yeast homologues of mammalian translation initiation factor 3 subunits p66 (eIF3d) and p48 (eIF3e), respectively, are required for stable association of eIF3 subunits. J Biol Chem 277, 2360–2367. 11705997 10.1074/jbc.M107790200

[10] Yahalom A , Kim TH , Winter E , Karniol B , von Arnim AG and Chamovitz DA (2001) Arabidopsis eIF3e (INT‐6) associates with both eIF3c and the COP9 signalosome subunit CSN7. J Biol Chem 276, 334–340. 11029466 10.1074/jbc.M006721200

[11] Zeng L , Wan Y , Li D , Wu J , Shao M , Chen J , Hui L , Ji H and Zhu X (2013) The m subunit of murine translation initiation factor eIF3 maintains the integrity of the eIF3 complex and is required for embryonic development, homeostasis, and organ size control. J Biol Chem 288, 30087–30093. 24003236 10.1074/jbc.M113.506147 PMC3798477

[12] Koyanagi‐Katsuta R , Akimitsu N , Hamamoto H , Arimitsu N , Hatano T and Sekimizu K (2002) Embryonic lethality of mutant mice deficient in the p116 gene. J Biochem 131, 833–837. 12038979 10.1093/oxfordjournals.jbchem.a003172

[13] Gildea DE , Luetkemeier ES , Bao X , Loftus SK , Mackem S , Yang Y , Pavan WJ and Biesecker LG (2011) The pleiotropic mouse phenotype extra‐toes spotting is caused by translation initiation factor Eif3c mutations and is associated with disrupted sonic hedgehog signaling. FASEB J 25, 1596–1605. 21292980 10.1096/fj.10-169771 PMC3079303

[14] Asano K , Merrick WC and Hershey JW (1997) The translation initiation factor eIF3‐p48 subunit is encoded by int‐6, a site of frequent integration by the mouse mammary tumor virus genome. J Biol Chem 272, 23477–23480. 9295280 10.1074/jbc.272.38.23477

[15] Marchetti A , Buttitta F , Miyazaki S , Gallahan D , Smith GH and Callahan R (1995) Int‐6, a highly conserved, widely expressed gene, is mutated by mouse mammary tumor virus in mammary preneoplasia. J Virol 69, 1932–1938. 7853537 10.1128/jvi.69.3.1932-1938.1995 PMC188811

[16] Mack DL , Boulanger CA , Callahan R and Smith GH (2007) Expression of truncated Int6/eIF3e in mammary alveolar epithelium leads to persistent hyperplasia and tumorigenesis. Breast Cancer Res 9, R42. 17626637 10.1186/bcr1742 PMC2206715

[17] Mayeur GL and Hershey JW (2002) Malignant transformation by the eukaryotic translation initiation factor 3 subunit p48 (eIF3e). FEBS Lett 514, 49–54. 11904180 10.1016/s0014-5793(02)02307-4

[18] Bandyopadhyay A , Matsumoto T and Maitra U (2000) Fission yeast Int6 is not essential for global translation initiation, but deletion of int6(+) causes hypersensitivity to caffeine and affects spore formation. Mol Biol Cell 11, 4005–4018. 11071923 10.1091/mbc.11.11.4005 PMC15053

[19] Akiyoshi Y , Clayton J , Phan L , Yamamoto M , Hinnebusch AG , Watanabe Y and Asano K (2001) Fission yeast homolog of murine Int‐6 protein, encoded by mouse mammary tumor virus integration site, is associated with the conserved core subunits of eukaryotic translation initiation factor 3. J Biol Chem 276, 10056–10062. 11134033 10.1074/jbc.M010188200

[20] Yen HC and Chang EC (2003) INT6–a link between the proteasome and tumorigenesis. Cell Cycle 2, 81–83. 12695651

[21] Shalev A , Valasek L , Pise‐Masison CA , Radonovich M , Phan L , Clayton J , He H , Brady JN , Hinnebusch AG and Asano K (2001) Saccharomyces cerevisiae protein Pci8p and human protein eIF3e/Int‐6 interact with the eIF3 core complex by binding to cognate eIF3b subunits. J Biol Chem 276, 34948–34957. 11457827 10.1074/jbc.M102161200

[22] Rencus‐Lazar S , Amir Y , Wu J , Chien CT , Chamovitz DA and Segal D (2008) The proto‐oncogene Int6 is essential for neddylation of Cul1 and Cul3 in Drosophila. PLoS One 3, e2239. 18493598 10.1371/journal.pone.0002239 PMC2375110

[23] Grzmil M , Whiting D , Maule J , Anastasaki C , Amatruda JF , Kelsh RN , Norbury CJ and Patton EE (2007) The INT6 cancer gene and MEK signaling pathways converge during zebrafish development. PLoS One 2, e959. 17895999 10.1371/journal.pone.0000959 PMC1978538

[24] Masutani M , Sonenberg N , Yokoyama S and Imataka H (2007) Reconstitution reveals the functional core of mammalian eIF3. EMBO J 26, 3373–3383. 17581632 10.1038/sj.emboj.7601765 PMC1933396

[25] Morris C and Jalinot P (2005) Silencing of human Int‐6 impairs mitosis progression and inhibits cyclin B‐Cdk1 activation. Oncogene 24, 1203–1211. 15558017 10.1038/sj.onc.1208268

[26] Morris C , Wittmann J , Jack HM and Jalinot P (2007) Human INT6/eIF3e is required for nonsense‐mediated mRNA decay. EMBO Rep 8, 596–602. 17468741 10.1038/sj.embor.7400955 PMC2002529

[27] Yen HC , Gordon C and Chang EC (2003) Schizosaccharomyces pombe Int6 and Ras homologs regulate cell division and mitotic fidelity via the proteasome. Cell 112, 207–217. 12553909 10.1016/s0092-8674(03)00043-6

[28] Hoareau Alves K , Bochard V , Rety S and Jalinot P (2002) Association of the mammalian proto‐oncoprotein Int‐6 with the three protein complexes eIF3, COP9 signalosome and 26S proteasome. FEBS Lett 527, 15–21. 12220626 10.1016/s0014-5793(02)03147-2

[29] Sha Z , Brill LM , Cabrera R , Kleifeld O , Scheliga JS , Glickman MH , Chang EC and Wolf DA (2009) The eIF3 interactome reveals the translasome, a supercomplex linking protein synthesis and degradation machineries. Mol Cell 36, 141–152. 19818717 10.1016/j.molcel.2009.09.026 PMC2789680

[30] Chen L , Uchida K , Endler A and Shibasaki F (2007) Mammalian tumor suppressor Int6 specifically targets hypoxia inducible factor 2 alpha for degradation by hypoxia‐ and pVHL‐independent regulation. J Biol Chem 282, 12707–12716. 17324924 10.1074/jbc.M700423200

[31] Chen L , Endler A , Uchida K , Horiguchi S , Morizane Y , Iijima O , Toi M and Shibasaki F (2010) Int6/eIF3e silencing promotes functional blood vessel outgrowth and enhances wound healing by upregulating hypoxia‐induced factor 2alpha expression. Circulation 122, 910–919. 20713899 10.1161/CIRCULATIONAHA.109.931931

[32] Skarnes WC , Rosen B , West AP , Koutsourakis M , Bushell W , Iyer V , Mujica AO , Thomas M , Harrow J , Cox T *et al* (2011) A conditional knockout resource for the genome‐wide study of mouse gene function. Nature 474, 337–342. 21677750 10.1038/nature10163 PMC3572410

[33] Mishina M and Sakimura K (2007) Conditional gene targeting on the pure C57BL/6 genetic background. Neurosci Res 58, 105–112. 17298852 10.1016/j.neures.2007.01.004

[34] Gillis LD and Lewis SM (2013) Decreased eIF3e/Int6 expression causes epithelial‐to‐mesenchymal transition in breast epithelial cells. Oncogene 32, 3598–3605. 22907435 10.1038/onc.2012.371

[35] Desnoyers G , Frost LD , Courteau L , Wall ML and Lewis SM (2015) Decreased eIF3e expression can mediate epithelial‐to‐mesenchymal transition through activation of the TGFbeta signaling pathway. Mol Cancer Res 13, 1421–1430. 26056130 10.1158/1541-7786.MCR-14-0645

[36] Zhou M , Sandercock AM , Fraser CS , Ridlova G , Stephens E , Schenauer MR , Yokoi‐Fong T , Barsky D , Leary JA , Hershey JW *et al* (2008) Mass spectrometry reveals modularity and a complete subunit interaction map of the eukaryotic translation factor eIF3. Proc Natl Acad Sci USA 105, 18139–18144. 18599441 10.1073/pnas.0801313105 PMC2587604

[37] Sun C , Todorovic A , Querol‐Audi J , Bai Y , Villa N , Snyder M , Ashchyan J , Lewis CS , Hartland A , Gradia S *et al* (2011) Functional reconstitution of human eukaryotic translation initiation factor 3 (eIF3). Proc Natl Acad Sci USA 108, 20473–20478. 22135459 10.1073/pnas.1116821108 PMC3251073

[38] Smith MD , Gu Y , Querol‐Audi J , Vogan JM , Nitido A and Cate JH (2013) Human‐like eukaryotic translation initiation factor 3 from Neurospora crassa. PLoS One 8, e78715. 24250809 10.1371/journal.pone.0078715 PMC3826745

[39] Sesen J , Cammas A , Scotland SJ , Elefterion B , Lemarie A , Millevoi S , Mathew LK , Seva C , Toulas C , Moyal EC *et al* (2014) Int6/eIF3e is essential for proliferation and survival of human glioblastoma cells. Int J Mol Sci 15, 2172–2190. 24481065 10.3390/ijms15022172 PMC3958844

[40] Rasmussen SB , Kordon E , Callahan R and Smith GH (2001) Evidence for the transforming activity of a truncated Int6 gene, *in vitro* . Oncogene 20, 5291–5301. 11536042 10.1038/sj.onc.1204624

[41] Chiluiza D , Bargo S , Callahan R and Rhoads RE (2011) Expression of truncated eukaryotic initiation factor 3e (eIF3e) resulting from integration of mouse mammary tumor virus (MMTV) causes a shift from cap‐dependent to cap‐independent translation. J Biol Chem 286, 31288–31296. 21737453 10.1074/jbc.M111.267294 PMC3173083

[42] Buchsbaum S , Morris C , Bochard V and Jalinot P (2007) Human INT6 interacts with MCM7 and regulates its stability during S phase of the cell cycle. Oncogene 26, 5132–5144. 17310990 10.1038/sj.onc.1210314

[43] Morris C , Tomimatsu N , Richard DJ , Cluet D , Burma S , Khanna KK and Jalinot P (2012) INT6/EIF3E interacts with ATM and is required for proper execution of the DNA damage response in human cells. Cancer Res 72, 2006–2016. 22508697 10.1158/0008-5472.CAN-11-2562 PMC3335344

[44] Neusiedler J , Mocquet V , Limousin T , Ohlmann T , Morris C and Jalinot P (2012) INT6 interacts with MIF4GD/SLIP1 and is necessary for efficient histone mRNA translation. RNA 18, 1163–1177. 22532700 10.1261/rna.032631.112 PMC3358639

