# Supplementary figures and images for: Eukaryotic translation initiation factor 3 (eIF3) subunit e is essential for embryonic development and cell proliferation
Source: FEBS Open Bio. 2018 Jul 5;8(8):1188–201. doi: 10.1002/2211-5463.12482 (PMC6070656; doi:10.1002/2211-5463.12482)

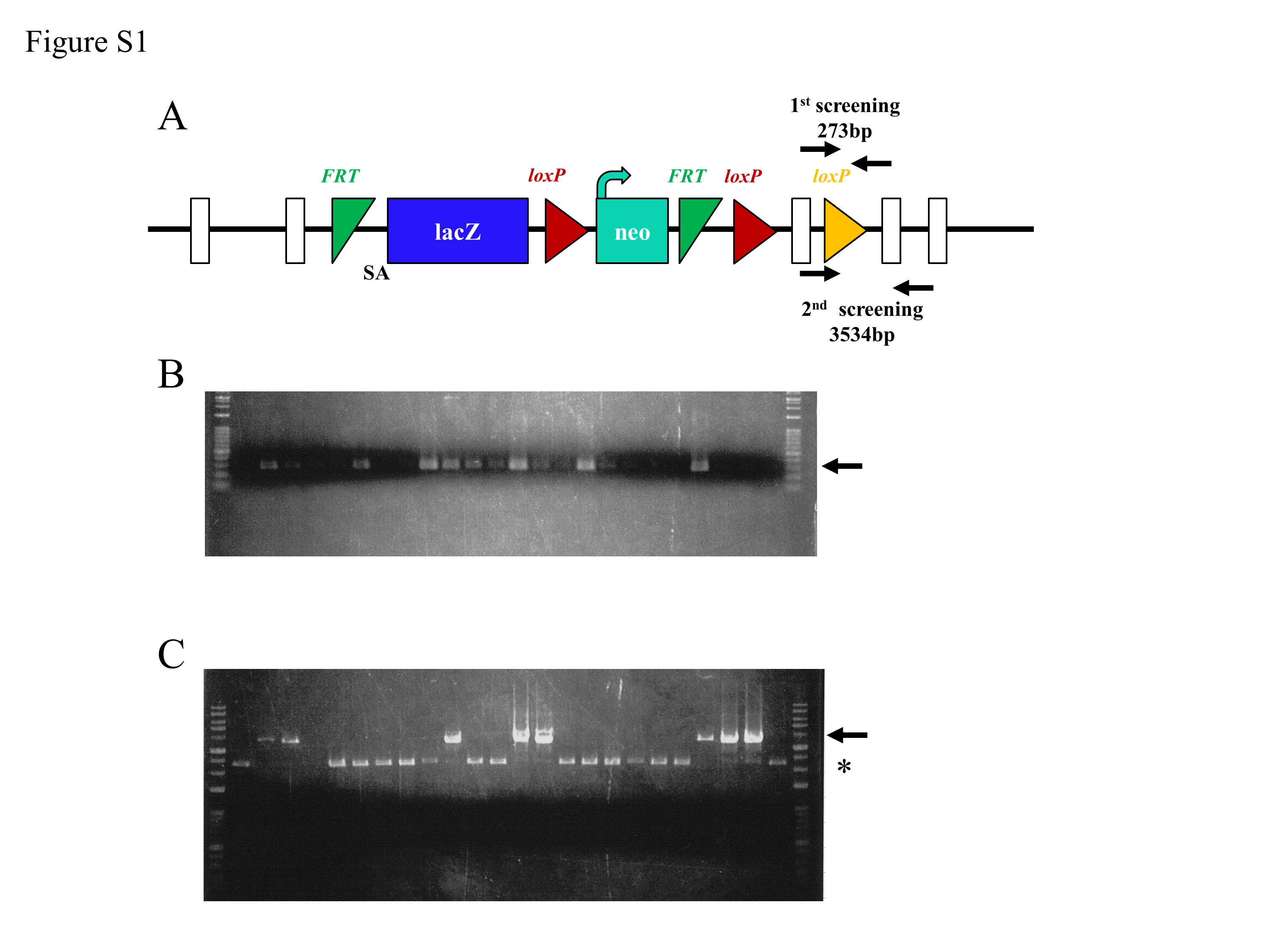

Supplement: Supplementary file 1 — Fig. S1. Screening of Int6/eIF3e‐targeted mouse ES cells by PCR. (A) The scheme for targeted ES cell screening by performing PCR. The homologous recombination allele and the two primer sets for the first and second screening are shown. (B) Representative results of PCR genotyping with the first set of screening primers. (C) Representative results of PCR genotyping with the second set of screening primers. Arrows indicate the predicted bands, and asterisk indicates nonspecific bands. [file FEB4-8-1188-s001.TIF]

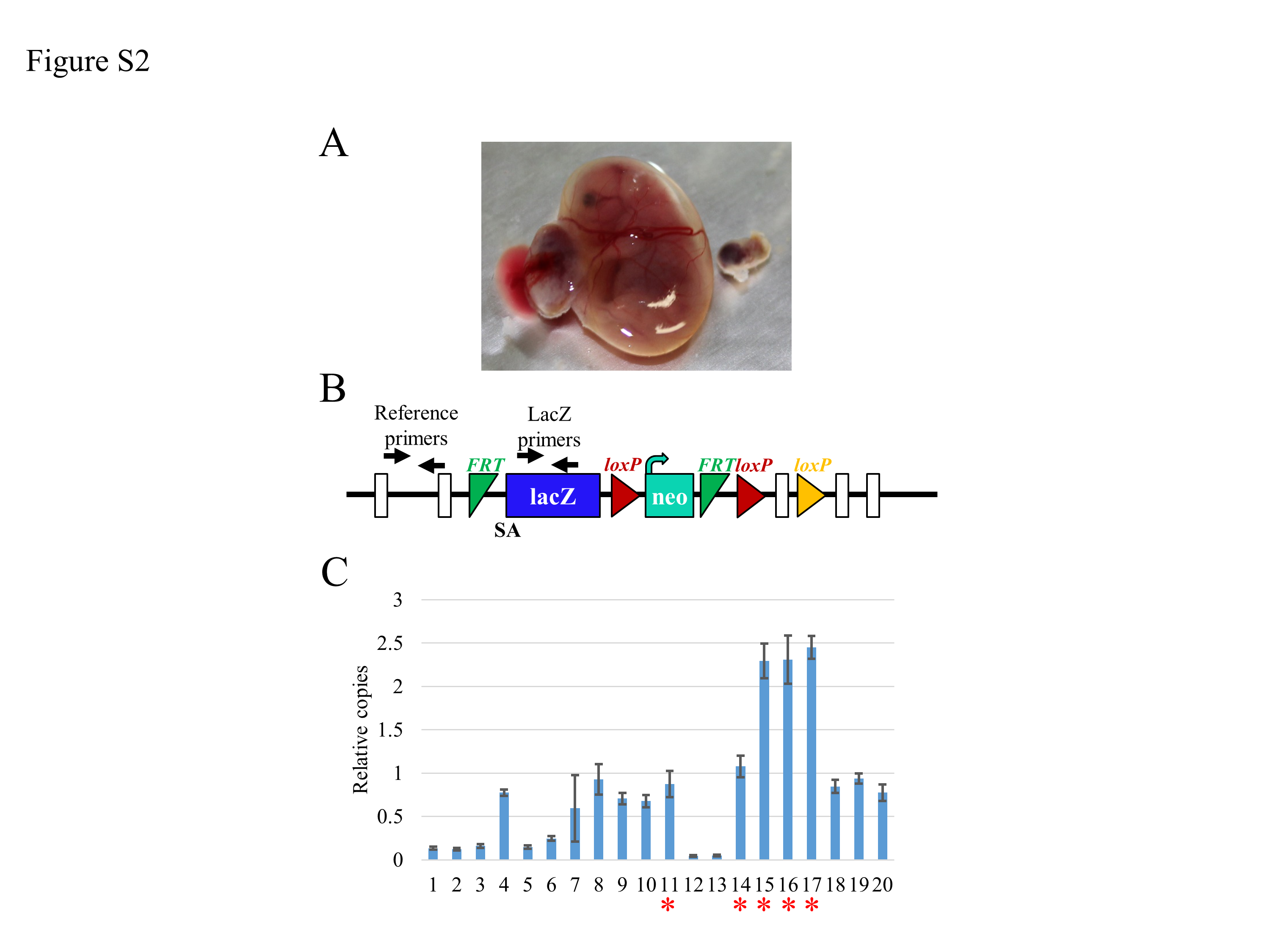

Supplement: Supplementary file 2 — Fig. S2. Copy number detection for genotyping of embryos. (A) Photo image of E13.5 embryos from eIF3e +/− intercrossing. (B) The genomic structure of eIF3e targeted homologous recombination allele, along with primers used for copy number assays, is shown. (C) Representative results of the copy number assay showing the copy number ratio (LacZ/eIF3e intron). Columns represent mean values with SD from triplicate samples. Asterisk indicates the sample with abnormality (extremely small or empty fetal membrane). [file FEB4-8-1188-s002.TIF]

Figure S3

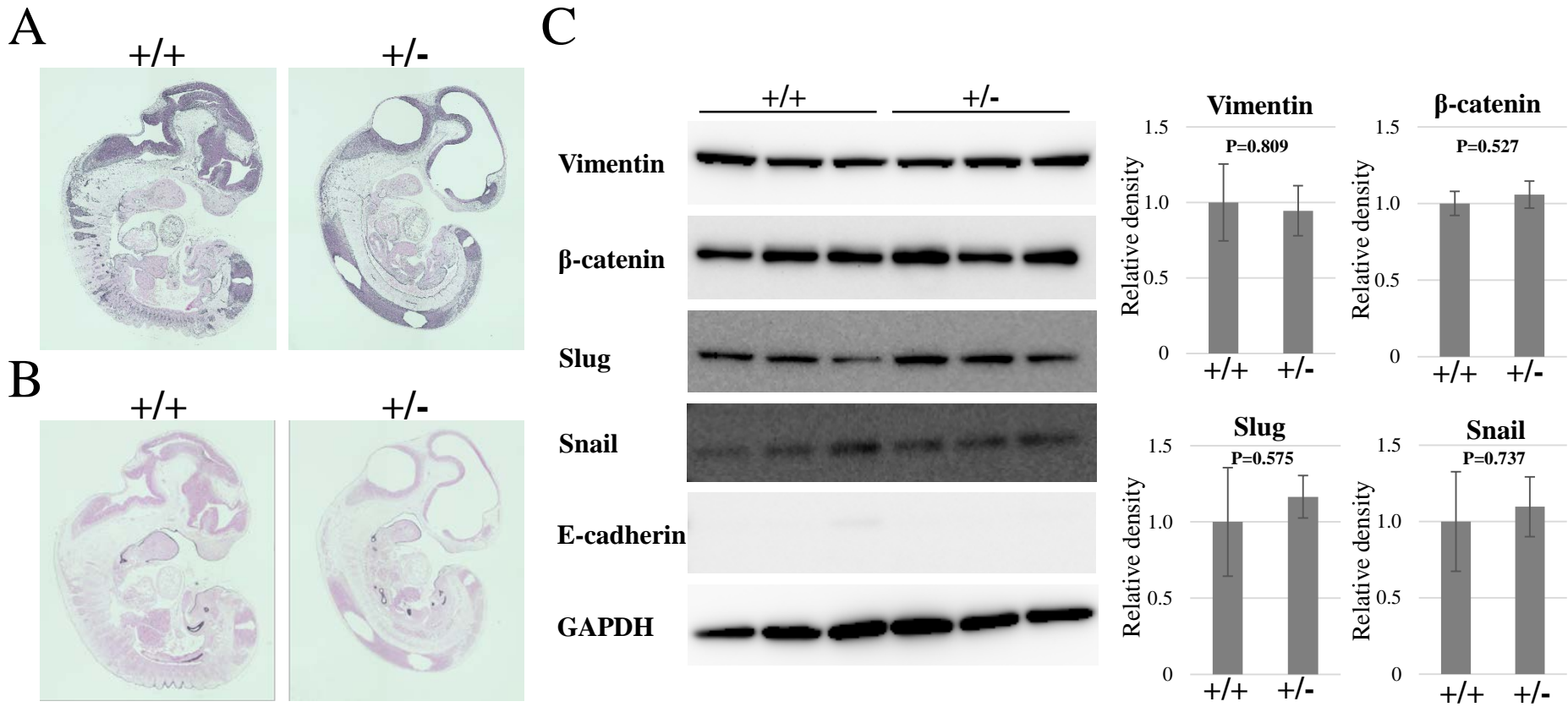

Supplement: Supplementary file 3 — Fig. S3. Expression of epithelial and mesenchymal markers in mice embryos and MEFs. Sagittal embryo sections show immunohistochemical analysis performed by antivimentin antibody (A, shown in black) and anti‐E‐cadherin antibody (B, shown in black) in eIF3e +/+ and eIF3e +/− at E10.5. (C) Western blotting of the indicated epithelial and mesenchymal marker proteins in the total cell lysates of eIF3e +/+ (+/+) and eIF3e +/− (+/−) MEFs from three independent clones. GAPDH was used as a loading control. Band densities were calculated from western blot results and shown by graphs (means ± standard deviation). P value was calculated using an unpaired two‐tailed Student's t‐test. [file FEB4-8-1188-s003.pdf]
