# Supplementary material for: Eukaryotic translation initiation factor 3 (eIF3) subunit e is essential for embryonic development and cell proliferation
Source: FEBS Open Bio. 2018 Jul 5;8(8):1188–201. doi: 10.1002/2211-5463.12482 (PMC6070656; doi:10.1002/2211-5463.12482)
Supplement: Supplementary file 4 — Table S1. Monoclonal and polyclonal antibodies used in western blot and immunohistochemistry. Table S2. Specific primers used in qRT‐PCR experiments. [file FEB4-8-1188-s004.docx]

**Supplementary Material**

**Table S1.** Monoclonal and polyclonal antibodies used in Western Blot and immunohistochemistry.

**For Western Blot**

| **Primary Ab (manufacturer)** | **Dilution** | **Secondary Ab (manufacturer)** | **Dilution** |
| --- | --- | --- | --- |
| Anti-eIF3a  (Cell signaling, Cat#2538) | 1/1000 | Anti-rabbit IgG HPR conjugate  (GE Healthcare, Cat#NA934) | 1/5000 |
| Anti-eIF3b  (Santa Cruz, Cat#sc-16377) | 1/1000 | Anti-goat IgG HPR conjugate  (Jackson Immuno Reserch,  Cat#[805-035-180](https://www.jacksonimmuno.com/catalog/products/805-035-180)) | 1/5000 |
| Anti-eIF3c  (Cell signaling, Cat#2068) | 1/1000 | Anti-rabbit IgG HPR conjugate  (GE Healthcare, Cat#NA934) | 1/5000 |
| Anti-eIF3d  (Santa Cruz, Cat#sc-271516) | 1/1000 | Anti-rabbit IgG HPR conjugate  (GE Healthcare, Cat#NA934) | 1/5000 |
| Anti-eIF3e  (Labmade, see reference [30]) | 1/5000 | Anti-rabbit IgG HPR conjugate  (GE Healthcare, Cat#NA934) | 1/5000 |
| Anti-eIF3h  (Cell signaling, Cat#3413) | 1/1000 | Anti-rabbit IgG HPR conjugate  (GE Healthcare, Cat#NA934) | 1/5000 |
| Anti-GAPDH  (Santa Cruz, Cat#sc-32233) | 1/5000 | Anti-mouse IgG HPR conjugate  (GE Healthcare, Cat#NA931) | 1/5000 |
| Anti-Vimentin  (Cell signaling, Cat#5741) | 1/5000 | Anti-rabbit IgG HPR conjugate  (GE Healthcare, Cat#NA934) | 1/5000 |
| Anti-β-Catenin  (Cell signaling, Cat#8480) | 1/1000 | Anti-rabbit IgG HPR conjugate  (GE Healthcare, Cat#NA934) | 1/5000 |
| Anti-Slug  (Cell signaling, Cat#9585) | 1/1000 | Anti-rabbit IgG HPR conjugate  (GE Healthcare, Cat#NA934) | 1/5000 |
| Anti-Snail  (Cell signaling, Cat#3879) | 1/1000 | Anti-rabbit IgG HPR conjugate  (GE Healthcare, Cat#NA934) | 1/5000 |
| Anti-E-Cadherin  (Cell signaling, Cat#3879) | 1/1000 | Anti-rabbit IgG HPR conjugate  (GE Healthcare, Cat#NA934) | 1/5000 |
| Anti-GFP  (MBL, Cat#M-048-3) | 1/5000 | Anti-mouse IgG HPR conjugate  (GE Healthcare, Cat#NA931) | 1/5000 |

**For immunohistochemistry**

| **Primary Ab (manufacturer)** | **Dilution** | **Secondary Ab (manufacturer)** | **Dilution** |
| --- | --- | --- | --- |
| Anti-Vimentin  (Cell signaling, Cat#5741) | 1/300 | Anti-rabbit IgG Biotin conjugate  (Vector Laboratories Inc., PK-6105) | 1/1000 |
| Anti-E-Cadherin  (Cell signaling, Cat#3879) | 1/100 | Anti-rabbit IgG Biotin conjugate  (Vector Laboratories Inc., PK-6105) | 1/1000 |

**Table S2.** Specific Primers used in quantitative RT-PCR experiments.

| **Genes** | **Forward (5’-3’)** | **Reverse (5’-3’)** |
| --- | --- | --- |
| *eIF3a* | CGGGAGGATCGATTCAGA | GATTCTTCTGCTGGTCCTCTTC |
| *eIF3b* | CCGCTACGTGGTTACCTCTG | CAGCCAGTAAGCATTGTCCA |
| *eIF3c* | GGGAACTACGGCAAACAGC | CGCTTCGGACAACTCTCTTT |
| *eIF3d* | TTCCAGAAGCAATTTGGAGTG | ACTGAGGAGTCTCGGGGTTT |
| *eIF3e* | CCAAATGTTGGCCATGAATA | AGCCAGAGTCTTGGGTTGC |
| *eIF3h* | GCAGCAGCAGAAACATCAGT | GCTCGCCTCGACTCTGTC |
| *gapdh* | GGGTTCCTATAAATACGGACTGC | CCATTTTGTCTACGGGACGA |
| 18S rRNA | GCAATTATTCCCCATGAACG | GGGACTTAATCAACGCAAGC |
